# Platelet indices as prognostic markers of vascular complications in type-2 diabetes mellitus

**DOI:** 10.6026/973206300223486

**Published:** 2026-06-30

**Authors:** Anita Omhare, Kanhaiya Lal Mishra, Medha Misra, Preeti Verma, Sandeep Kumar, Prashant Kumar

**Affiliations:** 1 Department of Pathology, Dr. Bhimrao Ramji Ambedkar Government Medical College, Kannauj, Uttar Pradesh, India; 2 Department of Community Medicine, Dr. Bhimrao Ramji Ambedkar Government Medical College, Kannauj, Uttar Pradesh, India; 3 Department of Community Medicine, Autonomous State Medical College, Kaushambi, Uttar Pradesh, India; 4 Department of Multidisciplinary Research Unit, Dr. Bhimrao Ramji Ambedkar Government Medical College, Kannauj, Uttar Pradesh, India

**Keywords:** Mean platelet volume (MPV), platelet count (PC), platelet indices, type 2 diabetes mellitus (T2DM), vascular complications

## Abstract

With its rising incidence and substantial risk of microvascular and macrovascular consequences, diabetes mellitus (DM) is a serious health concern of global magnitude. Therefore, it is of interest to determine the distribution of platelet indices in type 2 diabetes mellitus (T2DM) patients with and without vascular complications and assess their potential as predictive biomarkers. The cross-sectional study was undertaken over 18 months, which included 107 diagnosed T2DM patients and 69 age and gender-matched healthy controls. Platelet count and MPV were significantly altered in diabetics, especially among those with complications, whereas PDW showed no significant difference and logistic regression displayed a significant correlation of abnormal platelet count (Adjusted OR: 2.95, 95% CI: 1.23-7.06; p=0.015) and MPV (Adjusted OR: 0.31, 95% CI: 0.10-0.92; p=0.036) with vascular complications. Platelet count and MPV are promising, economical biomarkers for the early prediction of vascular complications in T2DM patients and their frequent monitoring may lead to early disease detection, timely anagement and better clinical outcomes.

## Background:

Diabetes mellitus (DM) is a globally prevalent medical concern owing to its rising incidence and high risk of complications. According to The International Diabetes Federation (IDF) around 463 million individuals are estimated to be affected globally in 2019, the numbers are projected to escalate to 700 million by the year 2045 [1]. In India, diabetes mellitus has grown to epidemic proportions, with approximately 72 million people affected in 2021. Diabetes mellitus patients possess twice the risk of mortality as compared with general population, due to micro-and macro vascular complications of diabetes. Long-term negative effects on several organs, including the eyes, kidneys, heart and brain, are caused by microvascular consequences that include retinopathy, nephropathy and neuropathy and macrovascular effects, such as cardiovascular and cerebrovascular disease and stroke [2, 3]. DM is linked with a prothrombotic state, largely driven by consistent hyperglycaemias, dyslipidemia and insulin resistance, which collectively induce endothelial damage. Additionally, variations in platelet structure and increased platelet reactivity play a critical part in the vascular comorbidities observed in diabetic patients, with platelet indices particularly platelet count (PC), mean platelet volume (MPV), platelet distribution width (PDW) and platelet large cell ratio (P-LCR) serving as important markers [4, 5]. MPV measured using nematology analysers, reflects platelet size, activation and production and serves as a commonly used marker of platelet activity linked with heightened cardiovascular risk [6, 7]. PDW indicates platelet heterogeneity, while platelet large cell ratio (P-LCR) measures large platelets [5]. Platelet indices are readily available, inexpensive tools for the early identification of vascular comorbidities in diabetes and may serve as useful predictive markers [8, 9, 10, 11]. Therefore, it is of interest to evaluate the platelet indices of diabetic patients with and without complications to ascertain their potential as cost-effective predictors and to enable early identification and timely intervention.

## Methodology:

This cross-sectional investigation was undertaken using information acquired over the course of 18 months (July 2023 to February 2025) from the Department of Pathology in collaboration with the Department of General Medicine and the Department of Community Medicine, catering to a rural population in North India. The study included 107 patients diagnosed with Type 2 diabetes mellitus, defined as per the American Diabetes Association (ADA) guidelines [12], alongside with 69 age- and gender-matched controls. Subjects with history of chronic diseases (e.g., kidney disease, liver disease, or hematological disorders), those receiving antiplatelet therapy (aspirin or clopidogrel), patients with malignant conditions, pregnant women and those patients who did not grant consent were eliminated from the research. Demographic and clinical parameters were evaluated based on the data collected through a pre-tested and pre- designed questionnaire, including age, sex, occupation and socioeconomic status. It also recorded anthropometric measurements (height, weight, BMI) and clinical findings, along with assessment of signs and symptoms of diabetic complications and their progression. A detailed treatment history, including duration of diabetes, type of therapy and treatment compliance, was also documented. Retinopathy was defined as the presence of ≥2 microaneurysms and retinal hemorrhages and was assessed by fundus examination by direct ophthalmoscopy [13]. Diabetic nephropathy was characterized as the presence of microalbuminuria (30-300 mg/24h urinary protein excretion) or macroalbuminuria and was assessed by fully automatic biochemistry analyser (SELECTRA PRO M ™ Q line Biotech Limited). Neuropathy was diagnosed according to the clinical symptoms of hyperesthesia/paraesthesia/motor weakness or polyradiculopathy. Biochemical and hematological evaluation was conducted on venous blood samples drawn aseptically from the antecubital vein, with 2 mL in K2 EDTA tubes and 4 mL in plain tubes and analyzed within two hours to minimize *in vitro* changes. Complete hemogram, including PC, MPV and PDW, was performed using the automated hematology analyzer (ABBOTT®, CELL-DYN RUBY 100). HbA1c was estimated using an automated HPLC analyzer (BIORAD® D-10 Hemoglobin Testing System, Bio-Rad Laboratories India Pvt. Ltd.), while blood glucose and microalbumin levels were qunatified using a fully automated biochemistry analyzer (SELECTRA PRO M™, Q-line Biotech Limited).

## Data analysis:

Data were entered and interpreted using SPSS software (IBM, version 21.0). Descriptive statistics such as mean, standard deviation, cross- tabulations and frequency distributions were applied. Continuous variables were reported as mean ± standard deviation, while categorical variables were summarized using frequencies and percentages. The correlation of platelet parameters and vascular complications was assessed with binary logistic regression and the Mann-Whitney U test. A p-value <0.05 was determined to be statistically significant.

## Results:

The research comprised of 107 participants, with individuals aged between 18 and 70 years. The majority were aged 36-50 years (43.0%), followed by 51-65 years (40.2%), while 9.3% were aged 18-35 years and 7.5% were above 65 years (Figure 1 - see PDF). Of these, 65 (60.7%) were male and 42 (39.3%) were female, with most residing in rural areas (88.8%) and 11.2% from urban settings. BMI classification showed that 56.1% had normal BMI (18.5-25 kg/m^2^), 35.5% were overweight (25-30 kg/m^2^), 4.7% were obese (>30 kg/m ^2^) and 3.7% were underweight (<18.5 kg/m^2^). Hypertension was the most prevalent co-morbidity, present in 81 patients (75.7%), while 26 (24.3%) had no comorbidities. Regarding pharmacological management, 30 patients (28.0%) were not on any drug therapy, 24 (22.4%) were on monotherapy, 47 (43.9%) were receiving dual drug therapy and 6(5.6%) required triple drug therapy. Among the patients, 45 cases (42.1%) had one or more vascular complications including hypertension, neuropathies, diabetic foot, retinopathy, nephropathy, coronary artery disease, peripheral vascular disease, hypertriglyceridemia and hypercholesterolemia while 62 (57.9%) had none; of the 45 affected, 8 had both microvascular and macrovascular comorbidities, with the remainder having only microvascular involvement. The mean HbA1c was markedly elevated in cases (9.01±2.07) compared to controls (4.84±0.74; p<0.001). Mean platelet count and MPV differed significantly between cases (162000±114453 cells/cumm; 12.03±1.90 fL) and controls (200000±90849 cells/ cumm; 22.94±9.93 fL), whereas PDW showed no significant variance (17.71±2.85% versus 17.58±3.20 fL) (Table 1). Abnormal platelet count was more commonly reported in subjects with cardiovascular complications and emerged as a major independent predictor after adjustment (Adjusted OR: 2.95; p=0.015). In contrast, abnormal mean platelet count demonstrated a significant inverse correlation with cardiovascular complications (Adjusted OR: 0.31; p=0.036). Although mean platelet volume showed an association on univariate analysis, this was not significantly associated following adjustment (Table 2). Applied Binary logistic regression, p<0.05), Data presented as Mean ± Standard Deviation or n (%). p < 0.05 was considered statistically significant. OR: Odds Ratio; CI: Confidence Interval; Ref: Reference category. Adjusted OR is derived from binary logistic regression.

## Discussion:

Diabetes mellitus is a metabolic condition governed by persistent hyperglycemia and metabolic dysregulation, predisposing the individuals to microvascular and macrovascular complications [14]. Diabetes and its comorbidities result in an immense burden on our country's economy and health infrastructure and its increasing prevalence among rural population of India [15] urges us to seek for a readily available and affordable diagnostic tool for early identification of these complications. Thus, efforts are aimed towards exploring the relevance of platelet indices in the prompt detection of these complications, as their routine availability makes them a potentially valuable tool in patient care.In this investigation, the mean HbA1c levels among diabetic patients were markedly raised than that of controls (4.84 ± 0.74, p<0.001), highlighting poor glycemic control in a majority of patients. These observations coincide with those of Kshirsagar *et al.* [16], who also reported significantly raised HbA1c levels in diabetics with complications in contrast to those without (9.02 ± 1.64 vs. 8.24 ± 1.05; p<0.05), as well as Ahluwalia *et al.* [17], who also observed elevated HbA1c in subjects with complications compared to those without (8.33 ± 1.45 vs. 6.75 ± 0.61). The platelet indices evaluated in this investigation were platelet count, mean platelet volume (MPV) and platelet distribution width (PDW); which are routinely available, cost- effective hematological parameters derived from peripheral blood counts and can be repeatedly monitored and compared to assess their correlation with vascular comorbidities in diabetic patients. The investigation showed significantly lowered platelet counts in diabetic cases as opposed to controls (162,000 vs. 200,000 cells/µL, p<0.001), whereas Ahluwalia *et al.* (2024) [17], reported significantly higher platelet counts in diabetics with complications (3.12 ± 1.07 lakh/mm^3^) than in those without (2.45 ± 0.82 lakh/mm^3^). Likewise, Persy *et al.*[18] reported elevated platelet counts in patients with complications, although the variation was not statistically relevant. This disparity may be due to variations in study populations, methodology and the impact of other associated comorbidities. The study revealed significantly raised MPV in diabetics compared to controls (p=0.002), with further elevation in those with complications. This is consistent with Ahluwalia *et al.* [17], who reported increased MPV in patients with complications (12.74 ± 3.07 fL) as opposed to those without (8.65 ± 1.58 fL), as well as Persy *et al.* [18] and Kshirsagar *et al.* (2019) [16], who also observed elevated levels of MPV in diabetics, particularly in those with vascular complications. Brahmbhatt *et al.* [19] observed similar findings, showing a strong correlation between increased MPV and both microvascular and macrovascular problems. Taderegew *et al.* [20] also identified MPV as an independent prognosticator of microvascular sequela in type 2 diabetes mellitus. However, in contrast, the results on PDW yielded no significant variance among diabetics and controls (p=0.872). These observations are in line with the study by Kshirsagar *et al.* [16], who also documented that no relevant correlation exists between PDW and complications. Similarly Persy *et al.* [18] and Ahluwalia *et al.* [17] observed markedly higher PDW levels in diabetics with comorbidities in comparison to those without. This variation implies that the predictive role of PDW may alter based on population characteristics and sample sizes. The outcome of the study supports the growing evidence that MPV is a reliable marker of platelet activation and vascular complications associated with type 2 diabetes. Furthermore, the findings provide additional evidence regarding the utility of hematological parameters in identifying patients at increased risk for microvascular and macrovascular complications, especially in resource-limited settings where advanced diagnostic modalities may not be easily accessible. The study also contributes region-specific clinical data that may aid in improving early screening strategies, timely intervention and overall management of T2DM patients. Nevertheless, the impact of platelet count and PDW is still ambiguous, with results varying across studies. These differences emphasize the need for more extensive, multicentric research to standardize various platelet indices as potential prognostic tools in complications resulting from diabetes.

## Conclusion:

Abnormal platelet count and mean platelet volume were significantly associated with vascular complications in type 2 diabetes mellitus. As simple, inexpensive and routinely available hematological parameters, these indices show promise as early biomarkers for vascular risk assessment. Their incorporation into routine clinical monitoring may aid in the timely detection and management of high-risk patients.

## Figures and Tables

**Table 1:** HbA1c and Platelet indices among cases and controls (Applied Paired t- test, p<0.05)

	**Cases**		**Controls**		**Total**		**p-value**
	Mean	SD	Mean	SD	Mean	SD	
HbA1c	9.01	2.07	4.84	0.74	7.38	2.64	<0.001
Platelet count(cells/cumm)	162000	114453.28	200000	90849.17	177000	107198.99	<0.001
Mean platelet count (%)	12.03	1.9	22.94	9.93	16.3	62.17	0.002
Platelet distribution width (%)	17.71	2.85	17.58	3.2	17.66	2.98	0.872
*Data presented as Mean ±
Standard Deviation.
*p < 0.05 was considered
statistically significant.

**Table 2:** Univariate and multivariate analysis of platelet indices among patients with and without vascular complications

		**Cardiovascular complications**						
Platelet Index	Category	Present (n=45) n (%)	Absent (n=62) n (%)	p-value (Chi-square)Unadjusted OR (95% CI)	p-valueAdjusted OR (95% CI)	p-value
Platelet Count	Abnormal	26	25	2.03 (0.93-4.42)	2.95 (1.23-7.06)	
		-57.80%	-40.30%			
	Normal	19	37	0.074Ref	0.076Ref	0.015
		-42.20%	-59.70%			
Mean platelet Count	Abnormal	31	54	0.33 (0.12-0.87)	0.31 (0.10-0.92)	
		-68.90%	-87.10%			
	Normal	14	8	0.021Ref	0.025Ref	0.036
		-31.10%	-12.90%			
Mean platelet volume	Abnormal	22	43	0.42 (0.19-0.94)	0.48 (0.20-1.17)	
		-48.90%	-69.40%			
	Normal	23	19	0.032Ref	0.034Ref	0.107
		-51.10%	-30.60%			
